# The Health Literacy of Retinol Cream Usage Among Female Students in the Faculty of Artificial Intelligence at Al-Balqa Applied University

**DOI:** 10.7759/cureus.40223

**Published:** 2023-06-10

**Authors:** Ruba F Al-Sheyab, Nour A Negresh, Raya Marji, Husam A ALSalamat, Hamzeh A Hatamleh, Noura F Al-Nawaiseh, Waleed F Dabbas, Tariq N Al-Shatanawi

**Affiliations:** 1 Dermatology, Al-Balqa Applied University, Al-Salt, JOR; 2 Emergency Medicine, Al-Balqa Applied University, Al-Salt, JOR; 3 Pathology and Forensic Medicine, Al-Balqa Applied University, Al-Salt, JOR; 4 Internal Medicine, Al-Balqa Applied University, Al-Salt, JOR; 5 Biopharmaceutics and Clinical Pharmacy, The University of Jordan, Amman, JOR; 6 Neurosurgery, Al-Balqa Applied University, Al-Salt, JOR; 7 Public Health and Community Medicine, Al-Balqa Applied University, Al-Salt, JOR

**Keywords:** health awareness, skin care, dermatology, health literacy, retinol cream

## Abstract

Introduction: Considering people’s tendency to self-treatment, efforts to address the broad aspects of health literacy are extremely important. The study aimed to find out the level of health literacy related to the use of retinol creams among female undergraduate students of the Faculty of Artificial Intelligence at Al-Balqa Applied University.

Methods: This study adopted an analytical descriptive research methodology by designing and applying a questionnaire tool. The questionnaire consisted, after arbitration and testing its validity and stability, of 15 items. Each item represents one of the indicators for measuring the level of health literacy about the use of retinol creams. The sample of the study was a random sample of female students in the Faculty of Artificial Intelligence at Al-Balqa Applied University.

Results: A total of 221 undergraduate female students were enrolled in the study. The most important results were that the arithmetic mean for estimating the level of health culture among female students in the study on the use of retinol creams was 3.117 out of 5, with a relative weight percentage of 62.3% and an average total score on the general level of indicators of total health culture.

Conclusion: This study provided insights into the health literacy related to the use of retinol creams among female students. While the students demonstrated a high level of health education in some aspects, there were areas where their knowledge and practices could be improved. These findings can contribute to the development of educational programs and interventions aimed at promoting the safe and informed use of retinol creams among university students.

## Introduction

In recent times, the pursuit of youth and beauty has gained significant importance, as visible signs of aging like wrinkles, loss of firmness, enlarged pores, and skin pigmentation issues greatly impact women's quality of life. Considering people’s tendency to self-treatment, efforts to address the broad aspects of health literacy are extremely important [1]. Seeking the expertise of dermatologists has become crucial, as changes in the skin are among the most apparent signs of aging. It appears that the use of cosmeceutical products has been rising dramatically and at an unheard-of rate, particularly for topical cosmeceuticals [2,3]. However, there aren't many studies that detail the precise percentages of people from various social, educational, and national backgrounds who use cosmeceuticals. The global cosmeceuticals industry is anticipated to increase from USD 54.57 billion in 2022 to USD 96.23 billion by 2029 according to Fortune Business Insights. Cost-effective noninvasive methods of enhancing one's skin and hair appearance are increasingly sought after, primarily by women and less commonly by men and even children [4,5].

Skin aging encompasses the gradual accumulation of changes in the skin's various components due to the passage of time [6], and it can be classified into four categories: (1) intrinsic aging, characterized by smooth, pale, dry skin with fine wrinkles; (2) extrinsic aging, predominantly affecting the face and neck, caused by external factors such as sun exposure; (3) photo-aging, resulting from harmful UV light; and (4) hormonal aging, which leads to decreased collagen synthesis, skin thickness, hydration, and barrier function [7]. Exogenous aging, also known as photodamage resulting from UV rays and sunlight exposure, accelerates natural skin changes, including paler, thinner, and more transparent skin due to an increase in melanocyte size and a decrease in their numbers [8]. Dry skin is also a sign of premature aging, caused by reduced oil production from sebaceous glands [9]. The emergence of fine lines, wrinkles, sagging, and loss of elasticity can be attributed to decreased collagen production, leading to irregular elastin structure and weakened connective tissue [10].

To combat aging signs, individuals often turn to topical agents promoting cell renewal like retinoids and alpha hydroxy acids (AHAs) [11,12]. Tretinoin, a vitamin A derivative, effectively treats photoaging and improves skin thickness [13,14]. However, misuse of over-the-counter drugs can lead to dangerous implications and iatrogenic ill effects [15]. Prescription retinoids, while beneficial for acne and wrinkles, may cause irritation and other side effects [13]. They are also susceptible to degradation from heat, air, and light [16]. Research shows that not only sun exposure and UV radiation but also near-infrared and visible light contribute to wrinkle formation [17]. Therefore, topical treatments with retinoids and AHAs are recommended to minimize visible light-induced skin damage and rejuvenate photodamaged skin [18].

Considering the scarcity of evidence from Jordan, this study aims to investigate health literacy of retinol cream usage among college female students which could reflect on the level of knowledge among characteristically matched populations across the country and aid in the development of education strategies by different stakeholders for better community health.

## Materials and methods

To achieve the general objective of the study, a descriptive research approach was employed, utilizing both qualitative and quantitative methods. The qualitative analytical method involved reviewing the literature to define the research subject, problem, and conceptual dimensions. The quantitative analysis method involved collecting field information and data for statistical analysis. Although the study was conducted at one research site, the chosen randomized, double-blind design and representative sample aimed to address limitations. However, it is acknowledged that a longer study duration and larger sample size could have strengthened the conclusions.

Study population

The study was conducted at Al-Balqa Applied University in Jordan, specifically among female students of the Faculty of Artificial Intelligence aged 18-21 who represent the public female community with non-medical backgrounds. The research period spanned from January to April 2023, employing a randomized, parallel study design.

Ethical approval

Consent was obtained or waived by all participants in this study. Approval was obtained from the Faculty at Al-Balqa Applied University to distribute the study questionnaire. The study adhered to current Good Clinical Practice (cGCP) guidelines and the principles of the Declaration of Helsinki, overseen by a board-certified dermatologist. Verbal consent to participate and to publish the findings in the study was obtained from all participating students upon sufficient explanation of the research objectives. Participants enrolled voluntarily, and they were given the option to withdraw from the study. No personal information was obtained from the participating students.

Study tool

The primary data was collected using a paper-based and self-filling questionnaire which was distributed by the research team in person after a proper introduction about the research objective and obtaining consent. The questionnaire was developed based on study variables, measures, and relevant literature. The final questionnaire comprised 15 items each assessed by a 5-point Likert scale, validated for validity and statistical stability.

Validity of the study tool

To ensure the validity of the study tool, faculty members from Jordanian universities were approached for their opinions and input regarding the tool's suitability, item clarity, and alignment with the intended dimensions. Their feedback and suggestions led to modifications in the wording of certain items. To assess the tool's validity, Pearson correlation coefficients were calculated for each item and the overall tool. The results of the validity test, presented in Table 1, revealed that all coefficient values were statistically significant at a significance level of 0.01, indicating a high level of significance. Furthermore, all Pearson correlation coefficients for the items exhibited positive values greater than 0.3, demonstrating a strong constructive validity of the questionnaire. This confirms that the questionnaire effectively measures the intended constructs, as indicated by the extracted Pearson correlation coefficients. By incorporating the faculty members' input and employing statistical analysis, the study tool's validity was established, enhancing the overall reliability and accuracy of the research findings.

**Table 1:** The results of the validity test of the study tool at the level of the paragraphs of the tool in a manner ** The correlation is statistically significant at a significant level of 0.001

Paragraph NO	Paragraph link to dimension		Paragraph link to the tool	
	Pearson Correlation	Sig. (2-tailed)	Pearson Correlation	Sig. (2-tailed)
1	.592(**)	0.00	.482(**)	0.00
2	.493(**)	0.00	0.128	0.00
3	.382(**)	0.00	.230(**)	0.00
4	.504(**)	0.00	.447(**)	0.00
5	.603(**)	0.00	.614(**)	0.00
6	.661(**)	0.00	.552(**)	0.00
7	.337(**)	0.00	.310(**)	0.00
8	.639(**)	0.00	.590(**)	0.00
9	.522(**)	0.00	.329(**)	0.00
10	.702(**)	0.00	.637(**)	0.00
11	.820(**)	0.00	.687(**)	0.00
12	.593(**)	0.00	.512(**)	0.00
13	.739(**)	0.00	.613(**)	0.00
14	.360(**)	0.00	.321(**)	0.00
15	.685(**)	0.00	.592(**)	0.00

Tool reliability

The reliability of the study tool was assessed using Cronbach's alpha coefficient. A stability coefficient value greater than or equal to 0.60 is considered statistically acceptable, with a higher value indicating increased reliability. The reliability coefficients were extracted for both the individual items and the overall tool to verify their reliability. The results of the reliability test, displayed in Table 2, demonstrated that the general reliability coefficient for the entire tool was 0.9001, indicating a reliability rate of 90%. The reliability coefficients for the individual items ranged from 0.826 (the lowest reliability percentage) to 0.857 (the highest reliability percentage). These findings indicate that the questionnaire has a high degree of reliability and can be confidently utilized in the field application of the study.

**Table 2:** The results of reliability at the level of the items of the tool on Cronbach's alpha

#	Item	Reliability coefficient Cronbach's alpha	Reliability %	degree of reliability
1	Item1	0.841	84%	high
2	Item 2	0.857	86%	high
3	Item 3	0.847	85%	high
4	Item 4	0.839	84%	high
5	Item 5	0.831	83%	high
6	Item 6	0.835	84%	high
7	Item 7	0.846	85%	high
8	Item 8	0.832	83%	high
9	Item 9	0.848	85%	high
10	Item 10	0.829	83%	high
11	Item 11	0.826	83%	high

Based on the latter validity and reliability tests, it can be concluded that the questionnaire used in the study exhibits both high validity and reliability. These results confirm the efficiency and accuracy of the questionnaire in measuring the intended constructs, further enhancing the credibility of the study findings.

Statistical analysis

All collected data were imported into a Microsoft Excel sheet (Microsoft, Washington, USA) to be sorted, cleaned, and coded. Statistical analyses were performed with SPSS Statistics version 28 (IBM Corp. Released 2021. IBM SPSS Statistics for Windows, Version 28.0. Armonk, NY: IBM Corp.). Graphical presentation of figures was carried out using Prism version 9.3.1 (GraphPad, San Diego, California, USA). The level of health culture regarding the use of retinol creams was reported as mean ± standard deviation.

## Results

**Figure 1:**
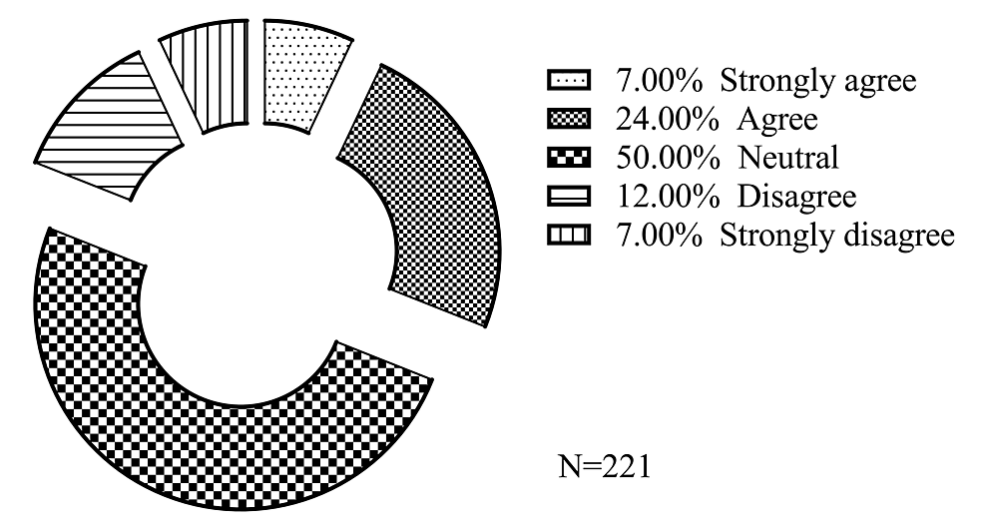
Overall distribution of participants’ perspectives on the importance of retinol cream

A total of 221 female students participated in the study upon formal consent. The age range of all students was 18-21 years old. All participating females were students at the Faculty of Artificial Intelligence at Al-Balqa Applied University between January and April 2023, the period of the study. Figure 1 presents the overall perspectives of participating students about the importance of retinol cream ranging from the neutral majority (50%) to various levels of agreement (31%).

To obtain the field results, the arithmetic means, standard deviations, and percentages were calculated for the responses of the research sample on the questionnaire, and in light of the quantitative statistical analysis, the researchers reached results related to the level of health culture regarding the use of retinol creams among female students of the Faculty of Artificial Intelligence at Al-Balqa Applied University at the macro level and at the level of health culture indicators.

The results are presented in Table 3. It is clear that the arithmetic mean of the research sample responses, at the overall level of the research tool, was 3.117 out of 5, with a total standard deviation of 0.423. The percentage weight of the research sample responses at the overall level was 62.3% at the overall level of the questionnaire. The arithmetic means for the degrees of the study sample's responses to the items of the questionnaire ranged between the two values: 2.118 as the lowest score and 3.376 as the highest score. The standard deviations ranged between 0.516 as the lowest standard deviation and 1.112 as the highest. According to the results, it was also found that the relative weights of the average scores of the study sample's approvals of the questionnaire items ranged between 42.4% as the lowest value and 67.5% as the highest.

**Table 3:** The arithmetic means, standard deviations, and the relative importance of the research sample responses to the paragraphs of the field study tool SD: standard deviation

#	Item	Mean score out of 5	SD	Relative significance	Rank	Degree
1	I know the importance of retinol creams	2.855	1.112	57.1%	13	Average
2	I have used retinol cream for the last 3 years	3.344	0.922	66.9%	4	Average
3	I have used retinol cream for acne	3.321	0.700	66.4%	7	Average
4	I have used retinol cream to prevent wrinkles	3.136	0.737	62.7%	11	Average
5	I have used retinol cream to treat the effects of the sun	3.348	0.791	67.0%	3	Average
6	My skin felt much better when using retinol creams	3.339	0.992	66.8%	5	Average
7	I use retinol creams without consulting a specialist	3.357	0.831	67.1%	2	Average
8	I read the leaflet for retinol creams carefully	2.498	0.822	50.0%	14	weak
9	I know the side effects of using a retinol cream	2.118	0.982	42.4%	15	weak
10	I am allergic to retinol creams	3.267	0.945	65.3%	8	Average
11	I do not use retinol creams as they cause burning and itching when applied	3.240	1.047	64.8%	9	Average
12	Prevention is better than cure. This phrase applies to the use of retinol creams	3.186	0.860	63.7%	10	Average
13	I advise my colleagues to use retinol creams to treat skin problems	3.376	1.101	67.5%	1	Average
14	I use a heavily moisturizing cream with retinol creams, as they cause dry skin	3.330	0.516	66.6%	6	Average
15	I stop using retinol creams when side effects such as peeling and redness appear.	3.041	0.831	60.8%	12	Average
Total		3.117	0.423	62.3%	Average	

## Discussion

Considering the growing evidence of the use of various cosmeceuticals to treat or self-treat different skin conditions, there is an immense need to address the level of knowledge about the potential risks and benefits of such readily available cosmeceuticals. The aim of this study was to assess the level of health literacy related to the use of retinol creams among female students at Al-Balqa Applied University.

The findings of the quantitative analysis revealed several results related to the students' level of health literacy. It was found that the highest average score was obtained in the item concerning providing and exchanging advice related to retinol cream use, indicating a high level of health education among the students in this aspect. This result aligns with previous studies recommending the prioritization of retinol creams used for skin problems [19]. However, the study also found that a moderate percentage of students reported using retinol creams without consulting a doctor.

Out of 318 Jordanian women surveyed in a previous study, 60.7% used skin-lightening products for various reasons including a preference for lighter skin and treating hyperpigmentation disorders believing lighter skin influenced self-esteem, beauty, youth, marriage, and employment [20]. Additionally, the use of retinol creams to treat sun effects and improve skin condition was also estimated at a moderate level. These findings are consistent with previous studies that highlighted the effectiveness of retinol creams in addressing sun damage and improving skin quality [21]. The study further revealed that students reported feeling a significant improvement in their skin when using retinol creams, which supports previous research findings [22]. The use of moisturizing creams alongside retinol creams to combat skin dryness was also observed at an average level among the students. This result is consistent with previous findings on the combination of (retinol 0.2%/LR2412 2%) versus tretinoin cream 0.025% in women with photobleached skin [23]. The latter study showed that both products significantly improved wrinkles, mottled pigmentation, pores, and photodamage. It was also shown from the results of the study that there are gradual adverse effects, but they are mostly mild. Moreover, retinol 0.2%/LR2412 2% is better tolerated and better perceived by women who follow rejuvenating procedures.

Regarding the use of retinol creams for specific purposes, such as treating acne and preventing the appearance of wrinkles, the study found that the students' level of use was estimated at an average degree. These findings are in line with previous studies that identified the effectiveness of retinol creams in addressing acne and reducing signs of aging [21,24].

The study also assessed the students' awareness of the risks associated with retinol cream use. It was found that students abstained from using retinol creams to avoid negative complications, such as burning and itching, at a moderate level. Moreover, students showed a moderate level of belief in the principle of prevention, indicating their awareness of the importance of taking precautions while using retinol creams. Nevertheless, in terms of medical and health knowledge about retinol creams, the study found that students had an average level of knowledge regarding the importance of retinol creams. However, their knowledge about the side effects of using these creams was relatively weak.

Based on the overall findings, the study concluded that the level of health education among female students regarding the use of retinol creams was moderate. These results align with previous studies that also reported moderate levels of health awareness among university students [25].

Limitations

Although this study was conducted in only one research site, we consider that the randomized, double-blind design chosen to conduct the study and the representative sample size allowed us to reduce final concerns about the limitations of this type of research, recognizing that the longer study and larger group size could have provided more robust conclusions.

## Conclusions

In conclusion, the field results of this study highlight the insufficient level of health education among female students in the study sample regarding the use of retinol creams. In light of these findings, the following recommendations are proposed. First, Jordanian universities should implement periodic annual programs to promote health awareness among female students in various medical fields, emphasizing body protection and prior medical health care to prevent the risks associated with using drugs and medicines without medical consultations. Second, dermatology and cosmetology specialists, such as doctors and pharmacists, should provide clear guidance and instructions to young girls on the proper and healthy use of retinol creams. Third, local and non-local pharmaceutical companies manufacturing moisturizers and creams should prioritize the development of effective policies, ensuring that instructions and contraindications are presented in a clear, understandable, and user-friendly manner. Lastly, it is recommended to conduct further studies in different social environments to enhance awareness and health literacy among young individuals, particularly girls, regarding the use of moisturizers and retinol creams.
